# Epicatechin Reduces Spatial Memory Deficit Caused by Amyloid-β25–35 Toxicity Modifying the Heat Shock Proteins in the CA1 Region in the Hippocampus of Rats

**DOI:** 10.3390/antiox8050113

**Published:** 2019-04-30

**Authors:** Alfonso Diaz, Samuel Treviño, Guadalupe Pulido-Fernandez, Estefanía Martínez-Muñoz, Nallely Cervantes, Blanca Espinosa, Karla Rojas, Francisca Pérez-Severiano, Sergio Montes, Moises Rubio-Osornio, Jorge Guevara

**Affiliations:** 1 Facultad de Ciencias Químicas, Benemérita Universidad Autónoma de Puebla, Puebla, Pue. PC. 72540, Mexico; alfonso.diaz@correo.buap.mx (A.D.); samuel_trevino@hotmail.com (S.T.); lupita.pulido.99@gmail.com (G.P.-F.); 2 Departamento de Bioquímica, Facultad de Medicina, Universidad Nacional Autónoma de México, Ciudad de México PC. 04510, Mexico; estefania.mtz17@gmail.com (E.M.-M.); nallely.co@gmail.com (N.C.); 3 Departamento de Bioquímica, Instituto Nacional de Enfermedades Respiratorias, SSA, Ciudad de Mexico, PC. 14269, Mexico; bespinosa1118@yahoo.com.mx; 4 Departamento de Ciencias de la Salud, Psicologia. Universidad del Valle de México, sede Sur., Ciudad de Mexico, PC. 04910, Mexico; karlacarmina_rojas@my.uvm.edu.mx; 5 Laboratorio de Neurofarmacología Molecular y Nanotecnología, Instituto Nacional de Neurología, SSA, Ciudad de Mexico, PC. 14269, Mexico; fperez@innn.edu.mx; 6 Departamento de Neuroquímica, Instituto Nacional de Neurología, SSA, Ciudad de Mexico, PC. 14269, Mexico; smontes@innn.edu.mx; 7 Laboratorio Experimental de Enfermedades Neurodegenerarivas, SSA, Ciudad de Mexico, PC. 14269, Mexico; ruomon@gmail.com

**Keywords:** reactive oxygen species, proinflammatory cytokines, Alzheimer’s disease

## Abstract

Alzheimer’s disease (AD) is a neurodegenerative disorder characterized by dementia and the aggregation of the amyloid beta peptide (Aβ). Aβ_25–35_ is the most neurotoxic sequence, whose mechanism is associated with the neuronal death in the Cornu Ammonis 1 (CA1) region of the hippocampus (Hp) and cognitive damage. Likewise, there are mechanisms of neuronal survival regulated by heat shock proteins (HSPs). Studies indicate that pharmacological treatment with flavonoids reduces the prevalence of AD, particularly epicatechin (EC), which shows better antioxidant activity. The aim of this work was to evaluate the effect of EC on neurotoxicity that causes Aβ_25–35_ at the level of spatial memory as well as the relationship with immunoreactivity of HSPs in the CA1 region of the Hp of rats. Our results show that EC treatment reduces the deterioration of spatial memory induced by the Aβ_25–35_, in addition to reducing oxidative stress and inflammation in the Hp of the animals treated with EC + Aβ_25–35_. Likewise, the immunoreactivity to HSP-60, -70, and -90 is lower in the EC + Aβ_25–35_ group compared to the Aβ_25–35_ group, which coincides with a decrease of dead neurons in the CA1 region of the Hp. Our results suggest that EC reduces the neurotoxicity induced by Aβ_25–35_, as well as the HSP-60, -70, and -90 immunoreactivity and neuronal death in the CA1 region of the Hp of rats injected with Aβ_25–35_, which favors an improvement in the function of spatial memory.

## 1. Introduction

Alzheimer’s disease (AD) is a neurodegenerative disorder that affects the senile population and is characterized clinically by loss of memory and difficulty in reasoning. At the histopathological level, neurofibrillary tangles (MNF) are formed by the hyperphosphorylated tau protein. Neuritic plaques (PNs) are formed by the amyloid-β peptide, which coexists with reactive astrogliosis and neuronal death in brain regions such as the cerebral cortex and the hippocampus (Hp) [1]. The amyloid-β (Aβ), the main component of the PNs, comes from the alternative hydrolysis of the amyloid precursor protein (PPA) [2]. There are several functional domains, characterized by a neurotoxic domain (25–35), of the amino acid sequence of Aβ that stand out. Several research groups propose the use of this neurotoxic domain as an experimental model to study AD [3,4].

The intrahippocampal injection of Aβ_25–35_ promotes the neurodegeneration accompanied by a deterioration in spatial memory [5,6]. Our previous results show that the undecapeptide generates an inflammatory response. This is evidenced by reactive astrogliosis and the release of proinflammatory cytokines such as interleukin-1 beta (IL-1β) and Tumor necrosis factor-alpha (TNF-α) [7], besides exacerbation of the production of reactive oxygen species (ROS) and lipid peroxidation, implying the generation of a chronic state of oxidative stress and cell death [4]. At the cellular level, the expression of heat shock proteins (HSPs), chaperones that participate in the assembly, transport, and degradation of proteins under both normal and stress conditions, has been demonstrated to be a cell survival mechanism [8]. Several reports indicate that the Aβ increases the expression of HSP-60, HSP-70, and HSP-90 as a protection mechanism against the toxicity of this peptide [9,10]. HSPs participate in cellular proteostasis and a reduction in the death of hippocampal neurons [11]. However, these reports indicate that the increase in these HSPs is not enough to reverse the neurotoxicity caused by the injection of Aβ into the rat Hp. Consequently, this causes neuronal and cognitive impairment [9]. 

In this sense, it is necessary to evaluate new molecules that, together with the HSP activity, can help to prevent or inhibit oxidative stress, the inflammatory response, and thus dementia. This could reverse the neurodegeneration induced by Aβ_25–35_ and be considered as a therapeutic alternative for AD. Recent reports indicate that epicatechin (EC), a flavonoid present in fruit and vegetables, has aroused great interest because of its beneficial antioxidant properties, also being used in the treatment and prevention of cancer as well as delaying aging [12,13]. The main biological activity of EC is the formation of protein complexes, the inhibition of free radicals, and the reduction of lipid peroxidation, making it an excellent antioxidant [14]. In addition, it can act as an anti-inflammatory by inhibiting cyclo-oxygenases and improving endothelial function [15,16].

Previously, it has been observed that EC treatment promotes the neuronal plasticity in the Hp and cortex, moreover improving spatial memory processes [16]. Also, EC prevents the toxicity induced by Aβ_25–35_ by reducing ROS and LP levels and reactive astrogliosis, and by not causing a deterioration of spatial memory [17,18]. However, it is not known how the anti-inflammatory and antioxidative activity of EC modifies the immunoreactivity of HSP-60, HSP-70, and HSP-90 in response to Aβ_25–35_ toxicity. The objective of this work is to evaluate the effect of EC administration on spatial memory, the oxidative-inflammatory response, and its relationship with HSPs after an intrahippocampal injection of Aβ_25–35_.

## 2. Materials and Methods 

### 2.1. Animals

Adult male Wistar rats (200–250 g, *n* = 60) were obtained from the vivarium of the faculty of medicine of the Universidad Nacional Autonoma de Mexico (UNAM). Animals were individually housed in a temperature- and humidity-controlled environment in a 12:12 h light-dark cycle with free access to food and water. The procedures described in this study were carried out in accordance with current national and international regulations for the use and care of laboratory animals of the Mexican Council of Animal Care (NOM-062-ZOO-1999), Guide of the National Institute of Health for the Care and Use of Laboratories, as well as Animals and Ethics Committee of the UNAM (UNAM-FMED-104-2017), in addition to minimizing the number of animals used for experimental purposes, which ensured generating the minimum possible pain.

### 2.2. Epicatechin Administration Protocol

Four experimental groups were considered for the study (15 rats per group): (1) Control (animals were given only sterile water), (2) Aβ_25–35_ [100 μM], (3) Epicatechin (EC) (200 mg/kg/day × 4 days), and (4) EC + Aβ_25–35_. The oral administration of EC started 1 day before the surgical injection and continued for 3 days more (4 days total). The treatment doses employed were chosen or calculated based on previous reports [18]. The Aβ_25–35_ peptide [100 μM] was dissolved in sterile water and the solution incubated at 37 °C for 24 h. The animals were then anesthetized with ketamine-xylazine (0.1 mL/100 g, i.p.) and placed in a stereotaxic frame (Stoelting Co. Wood Dale, Illinois, USA). The stereotaxic coordinates used to produce a bilateral lesion in the Hp were A: –4.2 mm from bregma, L: –2.0 mm from the midline, V: –2.2 below dura) [17]. Injections of Aβ_25–35_ or Control (1 µL) per side were administered for 5 min with a Hamilton syringe. After surgery, the animals were returned to their cages to recover.

### 2.3. Water Maze Spatial Task

After the treatment, the spatial memory was evaluated in the water maze (WM) at the Laboratory of Excitatory Amino Acids, Instituto Nacional de Neurología, INNN (Mexico City; Mexico), which consisted of a circular water pool (140 cm diameter and 80 cm high) that was filled with water to a height of 42 cm at 23 ± 24 °C and dyed with 0.01% white titanium oxide (TiO2). Four quadrants (N, S, E, or W) were traced into the pool. A platform of 20 cm diameter and 40 cm high, located at a constant position in the middle of one quadrant, was submerged 2 cm below the water’s surface. This procedure was repeated during 4 days of training. The animals (*n* = 15/group) were allowed to search for the platform for 90 s and were gently guided to it if they did not reach the target on their own. The time of 90 s was assigned as the maximum score. The parameter to be evaluated was the time to find the platform. The memory test was performed 2 days after training; in this test, the platform was removed from the pool. The test consisted of each of the animals performing a single try to find the place where the platform was. The parameter to be evaluated was the latency time at the first crossing, with the aim of evaluating if the animal memorized where the platform was located. The trials were recorded by a video camera mounted above the center of the pool (Sharp VL-WD450 U, SHARP Corporation, Osaka, Japan).

### 2.4. Quantifying IL-1β and TNF-α Cytokines

After the spatial memory test, the animals were decapitated (*n* = 8/group). The brain was removed from the cranial cavity of the animals. After, the Hp was extracted according to the protocol described by Diaz et al. [6] The hippocampal tissue was placed in a microcentrifuge tube with 1.5 mL of a phosphate buffer solution (PBS) (0.1 M; pH 7.4) at a temperature of 4 °C, to be liquefied for one minute at 100 rpm, with the help of a homogenizer. Afterward, homogenate hippocampal tissue was centrifuged at 12,500 rpm at 4 °C [19,20]. The supernatant was extracted from the centrifuged tissue and subsequently aliquoted in microtubes (200 mL) and stored at –70 °C. The supernatants were used for protein and proinflammatory cytokine measurements. The concentrations of IL-1*β* and TNF-*α* in the supernatants were quantified by an immunoassay procedure, as specified in the kit protocols (R&D Systems, Minneapolis, MN, USA). Samples were treated with a monoclonal antibody in precoated wells where the immobilized antibody bound to the protein. After washing away any unbound substances, an enzyme-linked specific antibody was added to the wells. The enzyme reaction yielded a blue product that turned yellow when the stop solution was added. Samples were read in a microplate reader at a wavelength of 450 nm where it was found that the intensity of the measured color was in proportion to the amount of each cytokine. 

### 2.5. Assay of Lipid Peroxidation 

The lipid-soluble fluorescent compounds were determined by means of the method established by Cuevas et al., and Perez Severiano et al. [17,21]. The supernatant obtained from the hippocampal tissue was mixed with chloroform-methanol (2:1) placed on ice and in a dark room. Subsequently, the chloroform phase was taken to quantify the fluorescence at an excitation of 370 nm and emission wavelengths of 430 nm on a Perkin Elmer LS50-B luminescence spectrometer (Waltham, MASS, USA). The fluorescent signal from the kit was adjusted to 140 fluorescence units (FU) with a standard quinine solution (0.001 mg/mL quinine in 0.05 M H_2_SO_4_). The results were expressed as relative FU (RFU) per mg of protein. [19].

### 2.6. Assay of Reactive Oxygen Species

We used 5 µL of the supernatant of the hippocampal tissue previously centrifuged, which was diluted in 9 volumes of TRIS and HEPES (40 mM). Subsequently, samples were incubated with 2’7’-dichlorodihydrofluorescein diacetate (DCFH-DA) (5 μM) [22] for one hour at 37 °C under constant agitation. The fluorescence signals were determined at 488 nm excitation and emission wavelengths of 525 nm (Perkin Elmer LS50-B luminescence spectrometer, Waltham, MASS, USA). The results were plotted as the mean of the DFC nm formed by mg of protein per minute. [20].

### 2.7. Determination of Superoxide Dismutase Activity

To evaluate the activity of superoxide dismutase (SOD), 20 μL of the supernatant of the hippocampal tissue previously centrifuged was used, which was incubated with reduced cytochrome-C solution (10 μM), sodium azide (NaN3, 10 μM), disodium ethylenediaminetetraacetic acid (EDTA, 10 mM), sodium bicarbonate (NaHCO3, 20 mM), xanthine (100 μM), and Triton X-100 with a pH of 10.2. To the reaction was added xanthine oxidase (0.1 mM EDTA) and the absorbance was determined at 550 nm. The activity of Zn-SOD was calculated as the total activity minus the activity measured in the presence of potassium cyanide (KCN, 1 mM) (Mn-SOD).

### 2.8. Histological Examination 

After the spatial memory test, the animals (*n* = 7 per group) were anesthetized with pentobarbital (40 mg/kg). Once the animal was in a state of hypnosis, the rib cage was opened and the pericardium was separated. Next, the vertex of the left ventricle was sectioned (with fine-tipped scissors) and a rigid cannula that was connected to the peristaltic pump was inserted, at a pressure of 80 mmHg that generated a continuous flow of 200 mL of PBS in the ascending aorta. The cannula was adjusted to the ventricle with the help of flat forceps. When the perfusion was started, the evacuation of the perfusion blood was facilitated by a cut in the right atrium. Once the perfusion with PBS was finished, perfusion was continued with 300 mL of 4% paraformaldehyde in a continuous flow and to the same pressure to ensure a better fixation of brain tissue. The brains were removed and postfixed in the same fixative solution for 48 h. The brain tissue was dehydrated and embedded in paraffin with the help of a histoquinet (Leica). Subsequently, the tissues were included in paraffin blocks and cut coronally (5 mm) with the help of a microtome (Leica), at the anterior temporal region level, in approximately 3.8 to 6.8 mm of bregma.

### 2.9. Immunofluorescence

The slides were deparaffinized and rehydrated using conventional histological techniques. They were then rinsed with a PBS (0.1 M; pH 7.4). Nonspecific binding sites were blocked by incubating in IgG-free 2% bovine serum albumin (Sigma) for 30 min at room temperature (RT). Afterward, specimens were permeabilized with 0.2% Triton X-100 (Sigma, St. Louis, MO, USA) for 10 min at RT. Sections were then incubated overnight at 4 °C to 8 °C with primary antibodies HSP-60, -70, -90 (1:200 dilution), and caspase-3 (1:100 dilution) (all primary antibodies were obtained from Santa Cruz Biotechnology Inc., CA, USA), followed by anti-rabbit Fluorescein Isothiocyanate (FITC) conjugated secondary antibodies and counterstained (1:100, Jackson Immuno Research Laboratories Inc., West Grove, PA, USA) with VectaShield-DAPI (Vector Labs., CA, USA) for nuclei staining. The photomicrographs were taken near the site of the injection using a fluorescence microscope (Leica Microsystems, Wetzlar, GmBH, Germany) and the number of immunoreactive cells to HSPs and caspase-3 were quantified in the CA1 subfield of the Hp. All counting procedures were made blindly by an expert in morphology.

### 2.10. Hematoxylin and Eosin (H&E) 

We evaluated the effect of EC on the neuronal loss caused by Aβ_25–35_ injection in the Hp by mean hematoxylin and eosin staining (H&E). The neurons were observed at 40× (Leica DM-LS, Leica Microsystems, Wetzlar, GmBH, Germany). Undamaged neurons were recognized as cells with round, blue nuclei and a clear perinuclear cytoplasm [19,20,21,22,23,24]. Damaged neurons were cells with changed nuclei (pyknosis, karyorrhexis, and karyolysis) and cytoplasmatic eosinophilia or loss of hematoxylin affinity. 

### 2.11. Statistical Analysis 

The results were expressed as the mean ± standard error (SE) for all experiments. Statistical analyses were done using variance analysis and multiple comparisons were made using Bonferroni post-test, considering *p* < 0.05 significant.

## 3. Results

### 3.1. Effect of Epicatechin Treatment on Spatial Memory in Rats Injected with Aß_25–35_ in the Hippocampus of Rats

**Figure 1:**
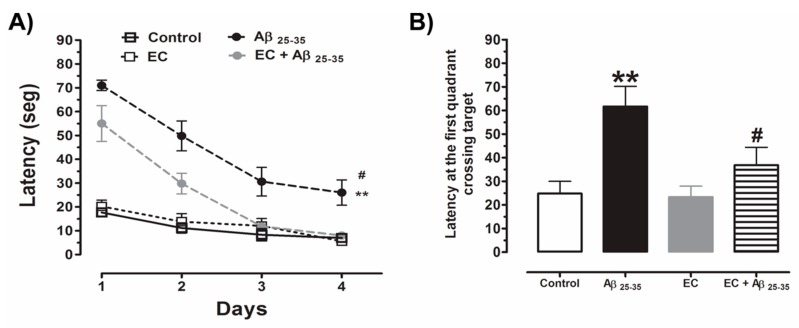
The administration of EC prevents the deterioration in spatial memory that induces the injection of Aβ_25–35_ in the rat Hp. The spatial training test and spatial memory test of rats administered with saline solution, epicatechin, Aβ_25–35,_ and epicatechin (EC) + Aβ_25–35_ were performed in the water maze. The parameters to be quantified were the latency time to find the escape platform (**A**) and latency of the first crossing in the objective quadrant (**B**). The values shown represent the standard error (SE) mean one-way ANOVA (** *p* < 0.01) comparing all groups with respect to the vehicle group. (# *p* < 0.05) compared the Aβ_25–35_ group versus EC + Aβ_25–35_ group.

The animals of each group performed the spatial training test in the WM, where the latency time to find the escape platform was quantified. The behavior of the animals of all the experimental groups to find the platform with respect to the days of training showed a progressive decrease in the latency time. The comparative analysis indicates that the group administered with Aβ_25–35_ showed a significantly longer latency time compared to the control group and the group administered only with EC on the different days that the training test was performed (one-way Analysis of Variance (ANOVA), *p* < 0.05). On the other hand, the group administered with EC + Aβ_25–35_ showed a latency time to find the platform lower in comparison with the group administered only with Aβ_25–35_ with a statistically significant difference that was observed from day 2 of training until the end of the test (One-way ANOVA, *p* < 0.05) (Figure 1A).

For the memory test, carried out two days after the learning test (Figure 1B), it is shown that the latency time at the first crossing of the target quadrant was significantly higher in the group treated with Aβ_25–35_ with respect to the other three experimental groups, respectively. Particularly, when comparing the group of Aβ_25–35_ with respect to the group EC + Aβ_25–35_, it is shown that the administration of EC significantly lowered the latency time at the first crossing of the white quadrant, suggesting a diminishing of cognitive damage caused by the neurotoxicity of the peptide into the Hp (one-way ANOVA, *p* < 0.05) (Figure 1A).

### 3.2. Effect of the Administration of Epicatechin on the Oxidative Response Induced by Aβ_25–35_ in the Hippocampus of Rats

**Figure 2:**
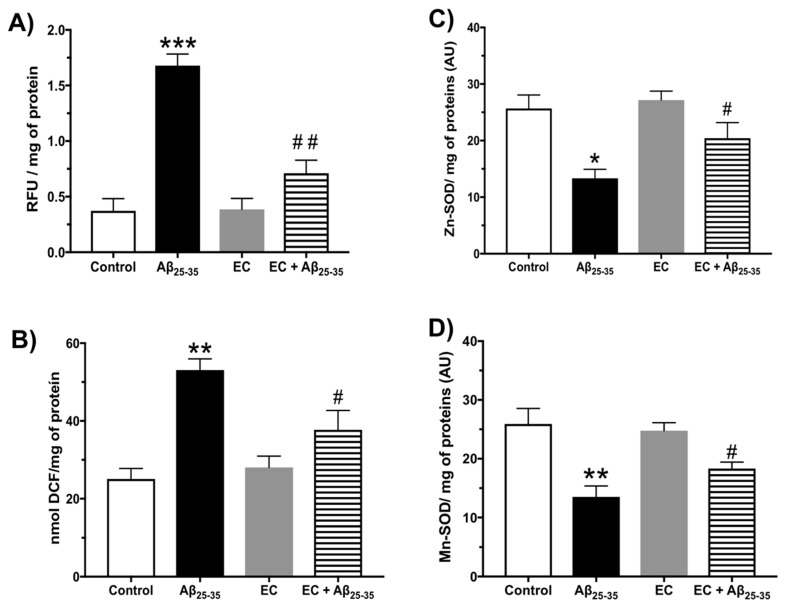
Effect of epicatechin on oxidative stress in the Hp of rats injected with Aβ_25–35_. (**A**) Reactive Oxygen Species (ROS) assay; (**B**) lipid peroxidation assay; (**C**) Zn-Superoxide activity assay; (**D**) Mn-Superoxide activity assay. The mean ± SE is plotted. Data were analyzed with one-way ANOVA and post-test Bonferroni test. (* *p* < 0.05; ** *p* < 0.01; and *** *p* < 0.001) comparing all groups with respect to the vehicle group. (# *p* < 0.05 and ## *p* < 0.01) compared the Aβ_25–35_ group versus EC + Aβ_25–35_ group.

The lipid lipoperoxidation data obtained from the Hp are shown in Figure 2A. The group injected with Aβ_25–35_ showed a significant fourfold increase compared to the basal levels of peroxidation in the control group. Treatment with EC + Aβ_25–35_ demonstrated its antioxidant effect by significantly decreasing (65%) the lipoperoxidation levels with respect to the group injected intrahippocampally with Aβ_25–35_ (one-way ANOVA with significance of *p* < 0.05), while the group treated only with EC did not show any considerable changes in lipid peroxidation when compared to the control group. The amount of ROS by 2,7-dichlorodihydrofluorescein found in the Hp is shown in Figure 2B. The statistical analysis reveals that the group injected with Aβ_25–35_ presented a significant increase of 59% regarding the control group. Likewise, when analyzing the data obtained from the hippocampi of the group treated with EC + Aβ_25–35_, there was a significant decrease of 28% in the levels of ROS in relation to the group injected only with Aβ_25–35_ (one-way ANOVA with a significance of *p* < 0.05). Regarding the group only injected with EC, the data obtained did not show a significant difference when compared with the control group. Figure 2C shows the enzymatic activity of Zn-SOD in the Hp. The Aβ_25–35_ group presented a decrement of 38% in SOD activity in relation to the vehicle group. In the same way, the SOD activity in the EC + Aβ_25–35_ group decreased by 25% with respect to the Aβ_25–35_ group (one-way ANOVA with a significance of *p* < 0.05), while between the control group and EC-treated group there were no differences. The Mn-SOD activity is shown in Figure 2D. The Aβ_25–35_ group and control group differed significantly by 35%. Likewise, when comparing the Mn-SOD activity in the EC + Aβ_25–35_ group in relation to the Aβ_25–35_ group, there was a significant change of 21% (one-way ANOVA with a significance of *p* < 0.05), while the EC group showed no differences regarding the control group.

### 3.3. Effect of Epicatechin on the Production of IL-1ß and TNF-α in the Hippocampus of Rats Injected with Aß_25–35_

**Figure 3:**
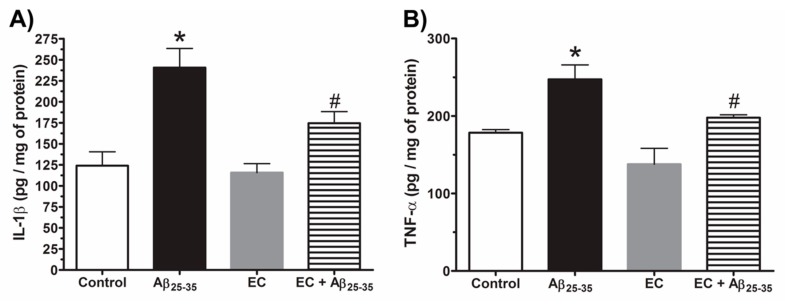
The epicatechin treatment decreases the concentration of proinflammatory cytokines in Hp of rats with Aβ_25–35_. (**A**) IL-1β in Hp and (**B**) TNF-α in Hp. The mean ± SE is plotted. Data were analyzed with one-way ANOVA and post-test Bonferroni test (* *p* < 0.05) comparing all groups with respect to the vehicle group. # *p* < 0.05 compared the Aβ_25–35_ group versus EC + Aβ_25–35_ group.

The concentrations of IL-1β and TNF-α were determined from a supernatant of the hippocampal tissues of each of the experimental groups (Figure 3). The concentration of IL-1β clearly shows that the intrahippocampal injection of Aβ_25–35_ was significantly higher compared to the control group. The EC treatment prevented the increase of the IL-1β in the EC + Aβ_25–35_ group (one-way ANOVA with a significance of *p* < 0.05), but the EC-treatment-only group did not show changes in relation to the control group. Similar behavior was observed in the TNF-α level in the Hp of the Aβ_25–35_ group, which was significantly higher compared to the rest of the experimental groups, and EC treatment prevented the exacerbation of the cytokine level after Aβ_25–35_ injection; however, TNF-α did not have changes in the EC group (one-way ANOVA with a significance of *p* < 0.05).

### 3.4. The Administration of Epicatechin Changes the Immunoreactivity of HSP-60, HSP-70, and HSP-90 in the Hippocampus of Rats Injected with Aß_25–35_

**Figure 4:**
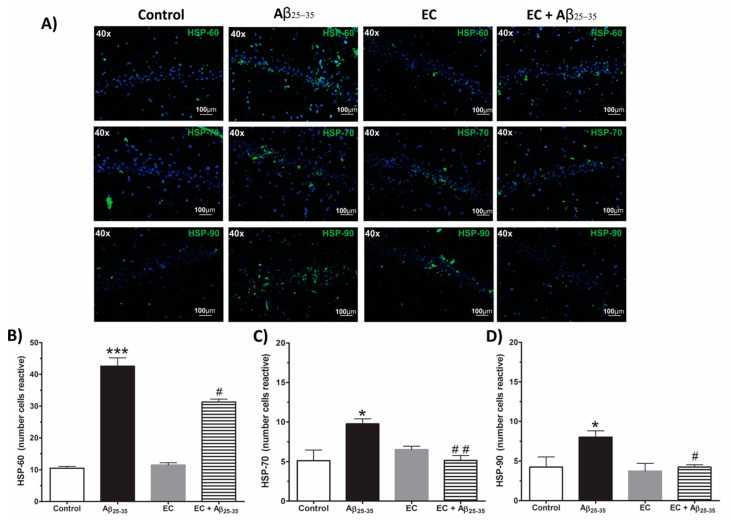
Effect of epicatechin on immunoreactivity of HSPs in CA1 subfields of the Hp of rats injected with Aβ_25–35_. In (**A**) we observed the immunoreactivity (green color) for HSP-60, HSP-70, and HSP-90 in the CA1 subfield of the Hp of rats with different treatments: control, Aβ_25–35_, EC, and EC + Aβ_25–35_. The epicatechin treatment decreases the number of immunopositive cells to HSP-60 (**B**) HSP-70 (**C**) and HSP-90 (**D**) in the Hp of rats injected with Aβ_25–35_ (**A**). The mean ± SE is plotted. Data were analyzed with one-way ANOVA and post-test Bonferroni test (* *p* < 0.05 and *** *p* < 0.001) comparing all groups with respect to the vehicle group. (# *p* < 0.05 and ## *p* < 0.01) compared the Aβ_25–35_ versus EC + Aβ_25–35_ groups.

To understand the reactivity of HSP in response to the toxicity of Aβ_25–35_ and the EC treatment in rat hippocampi, immunofluorescence was performed to identify the HSP-60, HSP-70, and HSP-90 in the Hp-CA1 region in each experimental group. The photomicrographs are shown in Figure 4A. Qualitative analysis indicates that the group injected with Aβ_25–35_ generated a greater immunoreactivity in the HSP (green color) in the CA1 region of the Hp compared to the control group. Particularly, HSP-60 showed a more intense label compared to HSP-90 and HSP-70. The treatment with EC in animals injected with Aβ_25–35_ caused a decrease in the immunoreactivity of the three chaperone proteins (green color). Specifically, the HSP-70 immunoreactivity decreased in greater proportion with respect to HSP-90 and HSP-60 in the CA1 region of the Hp.

The statistical analysis of the number of cells reactive to HSP-60, HSP-70, and HSP-90 (Figure 4B–D) indicates that the group with Aβ_25–35_ shows a significant increase in the number of immunoreactivity cells of 400%, 83%, and 200% for HSP-60, HSP-70, and HSP-90, respectively, in the CA1 region of the Hp, whereas the treatment with EC plus Aβ_25–35_ caused a significant reduction of 17%, 72%, and 58% for HSP-60, HSP-70, and HSP-90, respectively, in the CA1 region of the Hp (one-way ANOVA with significance of *p* < 0.05).

### 3.5. The Administration of Epicatechin Reduces the Immunoreactivity of Caspase-3 in the Hippocampus of Rats Injected with Aß_25–35_

**Figure 5:**
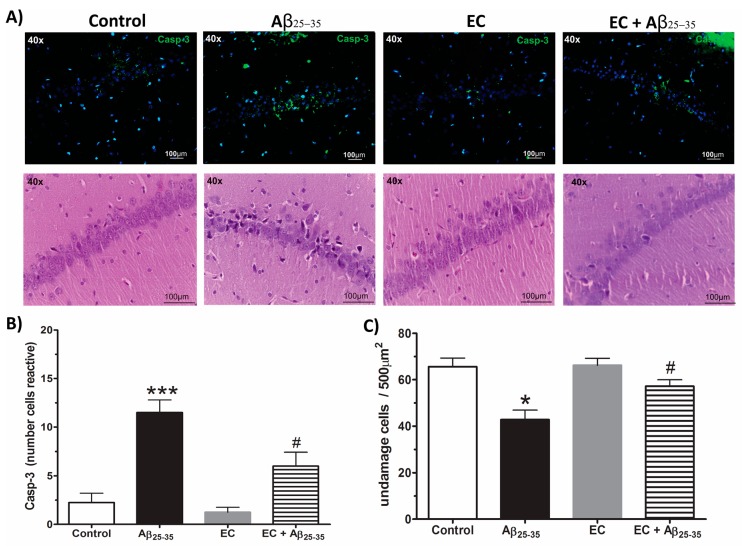
The epicatechin decreased the immunoreactivity of caspase-3 and the number of damaged cells in CA1 subfields of the Hp rats of injected with Aβ_25–35_. In (**A**) we observed the immunoreactivity for caspase-3 (green color) and the cells stained with H & E of the CA1 subfield of the Hp of rats control, Aβ_25–35_, EC, and EC + Aβ_25–35_ groups. The epicatechin treatment decreases the number of immunoreactive cells to caspase-3 (**B**) and a number of damaged cells in the CA1 Hp of rats injected with Aβ_25–35_ (**C**). The mean ± SE is plotted. Data were analyzed with one-way ANOVA and post-test Bonferroni test (* *p* < 0.05 and *** *p* < 0.001) comparing all groups with respect to the vehicle group. # *p* < 0.05 compared the Aβ_25–35_ and EC + Aβ_25–35_ groups.

We performed an immunohistochemistry for caspase-3 and hematoxylin and eosin staining to investigate whether EC reduces neuronal death. The photomicrographs are shown in Figure 5A. The images reveal that Aβ_25–35_ induces a greater immunoreactivity to caspase-3 (green color) in the cells of the CA1 region of the Hp, compared to the control group, while for the EC + Aβ_25–35_ group, there exists a lower marker to caspase-3, with respect to the group injected only with Aβ_25–35_. The number of immunoreactive cells to caspase-3 indicates that Aβ_25–35_ increases the reactivity to caspase-3 by 40% with respect to the control group. However, treatment with EC in animals with Aβ_25–35_ reduces the immunoreactivity by 45% in the cells of the CA1 region of the Hp (Figure 5B) (one-way ANOVA with a significance of *p* < 0.05). Hematoxylin and eosin staining show damage to the neurons through cytoplasmic changes of eosinophilia, in addition to the observed pyknosis, karyorrhexis, and karyolysis. This was more severe in the CA1 region of the Hp of the group treated with Aβ_25–35_ than in the EC + Aβ_25–35_ group. These indicators were not observed in the control and EC group (Figure 5B). Figure 5A shows the viable cell number in the CA1 subfield of the Hp. The number of viable neurons in the Aβ_25–35_ group was less compared with the control group (54%), whereas the number of viable cells in the EC + Aβ_25–35_ group was greater with respect to the Aβ_25–35_ group (65%) (one-way ANOVA with a significance of *p* < 0.05). The group treated only with EC showed no changes with respect to the control group. This indicates that EC decreases the neuronal death that Aβ_25–35_ induces in rat hippocampi.

## 4. Discussion

In the present work, we investigated whether treatment with EC is beneficial in combating the toxic effects of Aβ_25–35_ in the Hp. The results show an improvement in the spatial memory task, as well as a reduction in markers of oxidative stress and inflammation. Additionally, a decrease in the immunoreactivity of HSP-60, HSP-70, and HSP-90 was detected, strongly suggesting a positive regulating of the processes of protein aggregation and neurodegeneration. Therefore, the treatment with EC reduces the hippocampal death events induced by the injection of Aβ_25–35_. 

Catechins are a group of polyphenolic compounds belonging to the flavonoid class, present in high concentrations in a variety of plant-based fruits, vegetables, and beverages [25,26,27]. Particularly, EC shows protection against dementia, as well as diseases in which oxidative stress seems to be highly prevalent [13]. Oxidative stress is considered a key factor in the AD pathogenesis [28]. Aβ is responsible for promoting the generation of ROS, the oxidation of lipids to destabilize the neuronal membrane, and decreasing the activity of antioxidant enzymes such as SOD and catalase, which encourage the necrosis and apoptosis states [29]. Reports indicate that Aβ_25–35_ interacts with the cell membrane, altering the structure and function of the lipid bilayer and augmenting the concentration of ROS and lipid peroxidation, as shown in our results. In addition, the injection of Aβ_25–35_ into the Hp of rats produces a decrease in activity of SOD and catalase, indicating that the Hp is a brain region susceptible to oxidative and neuronal damage [30].

The interactions that Aβ_25–35_ has with the cell membrane induces ion channel dysfunction, which is associated with dysregulation of neuronal Ca^2+^. The intracellular increase of Ca^2+^ in neurons provokes vulnerability to the apoptotic processes [31]. In other reports, neuronal death is mainly associated with the generation of peroxidation products induced by Aβ_25–35_, such as 4-hydroxy-nonenal (4-HNE), which alters the glutamate transporter (GLT1). This causes the excessive entry of Ca^2+^ to activate the nitric oxide neuronal synthase (nNOS) responsible for catalyzing the formation of nitric oxide (NO) in neurons. The interaction of NO with the superoxide ion (O^2−^) promotes the formation of peroxynitrite (ONOO^−^) [7]. This reactive species alters the structure and function of proteins of the cytoskeleton and the mitochondria by means of a nitration or nitrosylation reaction, structures considered as cell damage markers which have been reported on several occasions by our research team [6]. The inflammatory response is a complementary mechanism of Aβ_25–35_ toxicity. The inflammation is mediated by the production and excessive release of proinflammatory cytokines, such as IL-1β and TNF-α. These cytokines are considered regulators of the intensity and duration of the inflammatory processes. TNF-induced cell death signaling is carried out by the TNF receptor (TNFR) [32]. Essentially, TNFR permits the recruitment of intracellular “death signaling inducing signaling complex” (DISC) proteins [33,34]. These proteins generate a scaffolding, so that recruitment is favored and caspase-8 is activated [35]. The freed, active caspase-8 then enzymatically processes procaspase-3, converting this executioner procaspase into active enzyme [35]. The activation of caspase-3 is essential for TNF-induced cell death, as it targets a latent DNase that degrades genomic DNA, thus causing apoptotic cell death, as was observed in the Aβ_25–35_ group [6,36]. On the other hand, IL-1 is a pivotal mediator of the inflammation that is expressed rapidly in response to neuronal injury, predominantly by microglia, and elevated levels markedly exacerbate the injury [37,38]. The associated mechanism to IL-1β release is the increase of proinflammatory molecules that results in rapidly recruited neutrophils, which cause tissue damage through the generation of free radicals, proteolytic enzymes, and more proinflammatory cytokines such as IL-1β and TNF-α [39]. The activation of both Nuclear Factor kappa B (NF-κB) and TNF-α triggers the activation of Mitogen-Activated Protein Kinase (MAPK)-related stress-activated Jun kinase (JNK) that induces cell death [40,41]. Studies have shown that increases in both IL-1β and TNF-α produce a ROS accumulation and cell death as we have observed in our results, where the intrahippocampal injection of Aβ_25–35_ showed a significant increase in the concentration of these cytokines compared to the control group [42]. Therefore, it is suggested that Aβ_25–35_ promotes an inflammatory response mediated by the production of proinflammatory cytokines, which could trigger the generation of ROS and the proliferation of glial cells, leading to reactive gliosis [43]. All these mechanisms of neurodegeneration cause damage to the structure and function of the limbic system, particularly in the Hp, a brain region that has a key role in the development of spatial memory, in such a way that a characteristic of the neurotoxicity of Aβ_25–35_ is the deterioration in learning and spatial memory, which is supported by a large number of publications [17,18,24,28,29].

On the other hand, unregulated ROS levels can activate oxidant stress sensor proteins, particularly the heat shock factor (HSF), a transcription factor responsible for increasing heat shock proteins (HSPs) because they react at the cellular level in response to environmental changes [44,45]. Under physiological conditions, HSPs act as molecular chaperones, preventing changes in the functional folding of proteins, while under stress conditions, they prevent protein aggregation [46]. Likewise, HSPs promote the regulation of specific signaling pathways that respond to inhibit apoptosis [47]. The neurotoxic changes caused by Aβ_25–35_ provoke conformational changes in HSF and induce the expression and synthesis of HSPs [48]. It is proposed that these proteins are activated as a neuroprotective mechanism. In this work, it is demonstrated that the increase in ROS and lipid peroxidation in response to the toxicity of Aβ_25–35_ promotes the increase in the immunoreactivity of HSP-60, HSP-70, and HSP-90 at 30 days postinjection with respect to the control group but does not prevent neuronal death.

Studies indicate that HSPs can be activated directly or indirectly. The ROS oxidize the thiol groups of proteins and cause the conformational change that activates the HSPs [49,50]. The in vitro and in vivo models indicate that each HSP performs specific functions in cells under conditions of oxidative stress. The HSP-90, being a folding chaperone, prevents the aggregation of the Aβ peptide (a necessary condition to cause neurotoxicity) and promotes its degradation. The increase in the expression of HSP-70 favors interaction with the intracellular Aβ, suggesting the sequestering of the peptide to avoid its interaction with other proteins and thus inhibiting its toxicity [9]. HSP-70 also activates microglia to synthesize and release cytokines and stimulate phagocytosis. There is evidence that HSP-70 promotes cell survival and reduces neuronal death [51]. It is also proposed that the overexpression of HSP-70 participates in inflammatory processes by reducing the levels of Cyclooxygenase (COX)-2 and the production of NO. In the same way, HSP-60, a mitochondrial chaperonin that is typically held responsible for the transportation and refolding of proteins from the cytoplasm into the mitochondrial matrix, also prevents the release of cytochrome-C in the mitochondria, the formation of apoptosome, and the activation of caspases, and therefore inhibits neuronal death. Reports indicate that Aβ impairs mitochondrial activity, however, HSP-60 prevents this damage and thus reduces the formation of ROS and promotes the native and functional conformation of SOD [52]. Our results indicate that all these chaperones act as damage biomarkers or neurosensors, suggesting their neuroprotective activity when increasing their immunoreactivity. These are active in different cellular compartments and interfere with oxidative, inflammatory processes and in the apoptotic signaling cascade that induces the toxicity of Aβ_25–35_ in the rat Hp.

The administration of EC increased spatial memory, decreased ROS concentrations and peroxidation levels, increased the enzymatic activity of SOD and catalase, and decreased IL-1β and TNF-α levels. It is proposed that EC captures oxidizing species and prevents lipid peroxidation, which results in blocking the neurotoxicity caused by Aβ_25–35_, therefore, the treatment of EC shows an improvement in spatial memory. It is also suggested that the catechol group in EC confers the ability to donate a hydrogen atom and therefore stabilizes the activity of free radicals, favoring antioxidant enzymes to help maintain redox balance [53]. Catechin treatments have demonstrated that they inactivate the Signal Transducer and Activator of Transcription 3 (STAT3) pathway, which plays a critical role in inflammation promotion, as well as being able to bind the p65 subunit of the transcription factor NF-κB and to inhibit cytokine and chemokines transcription stimulated by IL-1β and TNF-α. EC has also been shown to inhibit the aggregation of amyloidogenic proteins, including Aβ, present in AD [54]. Interestingly, in the presence of EC, it has been observed that the Aβ monomers adopt a new conformational form with an increased inter-center-of-mass distance with reduced β-sheet content [55,56]. This thereby affects its inclination to form fibril-prone states that otherwise increase the severity of Alzheimer’s disease [56]. At a peripheral level, EC at concentrations of 0.1–1 mM was found to inhibit nitrite formation and the IL-1β-induced expression of iNOS by blocking the nuclear localization of the p65 subunit of NF-κB [57]. Therefore, it is reasonable to hypothesize that at the central level and, particularly, in the CA1 hippocampal region, EC can use similar pathways. 

In addition, EC possesses the ability to directly or indirectly scavenge ROS by chemically reacting with ROS or by modulating pathways that regulate ROS scavenging compounds and enzymes, respectively [18]. EC can directly reduce ROS levels because the inhibitory strength of this compound is in the number of hydroxyl groups present [17]. In support of this idea, a [51] recent study confirmed that the o-catechol moiety of EC is essential for the direct detoxifying effects of the reaction with superoxide and hydrogen peroxide, or by blocking ROS generation [57,58,59]. EC also modulates the signaling of nuclear factor erythroid 2p45-related factor-2 (Nrf2), an important factor in cellular detoxification, proliferation, survival, and differentiation [60] that increases the synthesis and activity of antioxidant enzymes, such as SOD y catalase, correlating with our results. Likewise, chronic degenerative diseases have shown affectation on the oxidative phosphorylation machinery. Cytochrome c (Cyt-c) and cytochrome c oxidase (COX) catalyze the terminal and proposed rate-limiting step in the mitochondrial electron transport chain [60], thus their correct function is decisive in the dysregulations in mitochondrial respiration and cell signaling [51]. Since the oxidative phosphorylation machinery is suppressed in most of the degenerative diseases, it can be speculated that reactivation of mitochondrial function might be a strategy that interferes with the progress of the degenerative process. In this sense, EC stimulates mitochondrial respiration [51], significantly stimulating the expression of oxidative phosphorylation protein complexes. Also, EC can inhibit the decrease in mitochondrial succinate dehydrogenase activity, cytochrome c release, mitochondrial fragmentation, and cytochrome c oxidase protein levels [61], and thus inhibit the activation of ERK, caspase-3, and JNK, and the release of cytochrome c [51], diminishing cell death, as was observed. In the same way, oxidative and inflammation changes caused by the Aβ peptide can induce the unfolded, mitochondrial protein stress response. However, the expression of the nuclear-encoded HSP-60 mitochondrial chaperones prevents mitochondrial damage and reduces cell death [61]. It is suggested that EC modulates the cellular signaling cascade through the activation of different pathways, such as serine/threonine kinase 1 (AKT), PKC (protein kinase-C), and MAP kinases. All these pathways participate in the processes that mediate the activation of transcription factors and antiapoptotic signaling, promoting the regulation of the heat shock factor and consequently the downregulation of the HSPs. As far as we know, there are no reports that indicate the potential role of EC on the activity of HSP under conditions of cell stress.

## 5. Conclusions

This work provides information on the activity that this flavonoid induces on the reactivity of HSP and its consequences on the oxidative and inflammatory response to favor protection in the structure and function of hippocampal neurons and consequently an improvement in spatial memory. In this sense, it provides some data about flavonoid activity on the molecular mechanism for regulation of these chaperones, which assures cell survival in the Hp of animals intoxicated with Aβ_25–35_, and which could be soon applied to AD therapy. 

## References

[1] Hampel H., Vergallo A., Aguilar L.F., Benda N., Broich K., Cuello A.C., Cummings J., Dubois B., Federoff H.J., Fiandaca M. (2018). Precision pharmacology for Alzheimer’s disease. Pharmacol. Res..

[2] De Oliveira W.F., Dos Santos Silva P.M., Coelho L., Dos Santos Correia M.T. (2019). Biomarkers, Biosensors and Biomedicine. Curr. Med. Chem..

[3] Millucci L., Ghezzi L., Bernardini G., Santucci A. (2010). Conformations and biological activities of amyloid beta peptide 25-35. Curr. Protein Pept. Sci..

[4] Zussy C., Brureau A., Keller E., Marchal S., Blayo C., Delair B., Ixart G., Maurice T., Givalois L. (2013). Alzheimer’s disease related markers, cellular toxicity and behavioral deficits induced six weeks after oligomeric amyloid-beta peptide injection in rats. PLoS ONE.

[5] Maurice T., Lockhart B.P., Privat A. (1996). Amnesia induced in mice by centrally administered beta-amyloid peptides involves cholinergic dysfunction. Brain Res..

[6] Diaz A., Rojas K., Espinosa B., Chavez R., Zenteno E., Limon D., Guevara J. (2014). Aminoguanidine treatment ameliorates inflammatory responses and memory impairment induced by amyloid-beta 25-35 injection in rats. Neuropeptides.

[7] Diaz A., Mendieta L., Zenteno E., Guevara J., Limon I.D. (2011). The role of NOS in the impairment of spatial memory and damaged neurons in rats injected with amyloid beta 25-35 into the temporal cortex. Pharmacol. Biochem. Behav..

[8] Sottile M.L., Nadin S.B. (2018). Heat shock proteins and DNA repair mechanisms: An updated overview. Cell Stress Chaperones.

[9] Ortega L., Calvillo M., Luna F., Perez-Severiano F., Rubio-Osornio M., Guevara J., Limon I.D. (2014). 17-AAG improves cognitive process and increases heat shock protein response in a model lesion with Abeta25-35. Neuropeptides.

[10] Lackie R.E., Maciejewski A., Ostapchenko V.G., Marques-Lopes J., Choy W.Y., Duennwald M.L., Prado V.F., Prado M.A.M. (2017). The Hsp70/Hsp90 Chaperone Machinery in Neurodegenerative Diseases. Front. Neurosci..

[11] Liu Y., Zhang X. (2018). Heat Shock Protein Reports on Proteome Stress. Biotechnol. J..

[12] Natsume M. (2018). Polyphenols: Inflammation. Curr. Pharm. Des..

[13] Bernatova I. (2018). Biological activities of (-)-epicatechin and (-)-epicatechin-containing foods: Focus on cardiovascular and neuropsychological health. Biotechnol. Adv..

[14] Patel A.K., Rogers J.T., Huang X. (2008). Flavanols, mild cognitive impairment, and Alzheimer’s dementia. Int. J. Clin. Exp. Med..

[15] Jiang Z., Zhang J., Cai Y., Huang J., You L. (2017). Catechin attenuates traumatic brain injury-induced blood-brain barrier damage and improves longer-term neurological outcomes in rats. Exp. Physiol..

[16] Li Q., Zhao H.F., Zhang Z.F., Liu Z.G., Pei X.R., Wang J.B., Li Y. (2009). Long-term green tea catechin administration prevents spatial learning and memory impairment in senescence-accelerated mouse prone-8 mice by decreasing Abeta1-42 oligomers and upregulating synaptic plasticity-related proteins in the hippocampus. Neuroscience.

[17] Cuevas E., Limon D., Perez-Severiano F., Diaz A., Ortega L., Zenteno E., Guevara J. (2009). Antioxidant effects of epicatechin on the hippocampal toxicity caused by amyloid-beta 25-35 in rats. Eur. J. Pharmacol..

[18] Cruz-Gonzalez T., Cortez-Torres E., Perez-Severiano F., Espinosa B., Guevara J., Perez-Benitez A., Melendez F.J., Diaz A., Ramirez R.E. (2016). Antioxidative stress effect of epicatechin and catechin induced by Abeta25-35 in rats and use of the electrostatic potential and the Fukui function as a tool to elucidate specific sites of interaction. Neuropeptides.

[19] Trevino S., Aguilar-Alonso P., Flores Hernandez J.A., Brambila E., Guevara J., Flores G., Lopez-Lopez G., Munoz-Arenas G., Morales-Medina J.C., Toxqui V. (2015). A high calorie diet causes memory loss, metabolic syndrome and oxidative stress into hippocampus and temporal cortex of rats. Synapse.

[20] Diaz A., Trevino S., Guevara J., Munoz-Arenas G., Brambila E., Espinosa B., Moreno-Rodriguez A., Lopez-Lopez G., Pena-Rosas U., Venegas B. (2016). Energy Drink Administration in Combination with Alcohol Causes an Inflammatory Response and Oxidative Stress in the Hippocampus and Temporal Cortex of Rats. Oxid. Med. Cell. Longev..

[21] Perez-Severiano F., Rodriguez-Perez M., Pedraza-Chaverri J., Maldonado P.D., Medina-Campos O.N., Ortiz-Plata A., Sanchez-Garcia A., Villeda-Hernandez J., Galvan-Arzate S., Aguilera P. (2004). S-Allylcysteine, a garlic-derived antioxidant, ameliorates quinolinic acid-induced neurotoxicity and oxidative damage in rats. Neurochem. Int..

[22] Ali S.F., David S.N., Newport G.D. (1993). Age-related susceptibility to MPTP-induced neurotoxicity in mice. Neurotoxicology.

[23] Ramos-Martinez I., Martinez-Loustalot P., Lozano L., Issad T., Limon D., Diaz A., Perez-Torres A., Guevara J., Zenteno E. (2018). Neuroinflammation induced by amyloid beta25-35 modifies mucin-type O-glycosylation in the rat’s hippocampus. Neuropeptides.

[24] Diaz A., De Jesus L., Mendieta L., Calvillo M., Espinosa B., Zenteno E., Guevara J., Limon I.D. (2010). The amyloid-beta25-35 injection into the CA1 region of the neonatal rat hippocampus impairs the long-term memory because of an increase of nitric oxide. Neurosci. Lett..

[25] Kim H., Ramirez C.N., Su Z.Y., Kong A.N. (2016). Epigenetic modifications of triterpenoid ursolic acid in activating Nrf2 and blocking cellular transformation of mouse epidermal cells. J. Nutr. Biochem..

[26] Adler G., Hey W., Achenbach C., Kinzer A. (2003). Pretreatment interhemispheric EEG coherence is related to seizure duration in right unilateral electroconvulsive therapy. Neuropsychobiology.

[27] Wang Z.F., Liu J., Yang Y.A., Zhu H.L. (2018). A Review: The Anti-inflammatory, Anticancer, Antibacterial Properties of Four Kinds of Licorice Flavonoids Isolated from Licorice. Curr. Med. Chem..

[28] Nalivaeva N.N., Turner A.J. (2017). Role of Ageing and Oxidative Stress in Regulation of Amyloid-Degrading Enzymes and Development of Neurodegeneration. Curr. Aging Sci..

[29] Gulyaeva N.V., Bobkova N.V., Kolosova N.G., Samokhin A.N., Stepanichev M.Y., Stefanova N.A. (2017). Molecular and Cellular Mechanisms of Sporadic Alzheimer’s Disease: Studies on Rodent Models in vivo. Biochem. Biokhimiia.

[30] Kaminsky Y.G., Marlatt M.W., Smith M.A., Kosenko E.A. (2010). Subcellular and metabolic examination of amyloid-beta peptides in Alzheimer disease pathogenesis: Evidence for Abeta(25-35). Exp. Neurol..

[31] Maurice T., Strehaiano M., Duhr F., Chevallier N. (2018). Amyloid toxicity is enhanced after pharmacological or genetic invalidation of the sigma1 receptor. Behav. Brain Res..

[32] Tartaglia L.A., Rothe M., Hu Y.F., Goeddel D.V. (1993). Tumor necrosis factor’s cytotoxic activity is signaled by the p55 TNF receptor. Cell.

[33] Zhang M., Wang J., Jia L., Huang J., He C., Hu F., Yuan L., Wang G., Yu M., Li Z. (2017). Transmembrane TNF-alpha promotes activation-induced cell death by forward and reverse signaling. Oncotarget.

[34] Boldin M.P., Goncharov T.M., Goltsev Y.V., Wallach D. (1996). Involvement of MACH, a novel MORT1/FADD-interacting protease, in Fas/APO-1- and TNF receptor-induced cell death. Cell.

[35] Lim M.C.C., Maubach G., Sokolova O., Feige M.H., Diezko R., Buchbinder J., Backert S., Schluter D., Lavrik I.N., Naumann M. (2017). Pathogen-induced ubiquitin-editing enzyme A20 bifunctionally shuts off NF-kappaB and caspase-8-dependent apoptotic cell death. Cell Death Differ..

[36] Enari M., Sakahira H., Yokoyama H., Okawa K., Iwamatsu A., Nagata S. (1998). A caspase-activated DNase that degrades DNA during apoptosis, and its inhibitor ICAD. Nature.

[37] Simi A., Tsakiri N., Wang P., Rothwell N.J. (2007). Interleukin-1 and inflammatory neurodegeneration. Biochem. Soc. Trans..

[38] Morganti-Kossmann M.C., Rancan M., Stahel P.F., Kossmann T. (2002). Inflammatory response in acute traumatic brain injury: A double-edged sword. Curr. Opin. Crit. Care.

[39] Ferrara-Bowens T.M., Chandler J.K., Guignet M.A., Irwin J.F., Laitipaya K., Palmer D.D., Shumway L.J., Tucker L.B., McCabe J.T., Wegner M.D. (2017). Neuropathological and behavioral sequelae in IL-1R1 and IL-1Ra gene knockout mice after soman (GD) exposure. Neurotoxicology.

[40] De Smaele E., Zazzeroni F., Papa S., Nguyen D.U., Jin R., Jones J., Cong R., Franzoso G. (2001). Induction of gadd45beta by NF-kappaB downregulates pro-apoptotic JNK signalling. Nature.

[41] Tang Y., Lopez I., Baloh R.W. (2001). Age-related change of the neuronal number in the human medial vestibular nucleus: A stereological investigation. J. Vestib. Res. Equilib. Orientat..

[42] Diaz A., Limon D., Chavez R., Zenteno E., Guevara J. (2012). Abeta25-35 injection into the temporal cortex induces chronic inflammation that contributes to neurodegeneration and spatial memory impairment in rats. JAD.

[43] Benjamin I.J., McMillan D.R. (1998). Stress (heat shock) proteins: Molecular chaperones in cardiovascular biology and disease. Circ. Res..

[44] Driedonks N., Xu J., Peters J.L., Park S., Rieu I. (2015). Multi-Level Interactions Between Heat Shock Factors, Heat Shock Proteins, and the Redox System Regulate Acclimation to Heat. Front. Plant Sci..

[45] Gething M.J., Sambrook J. (1992). Protein folding in the cell. Nature.

[46] Calvillo M., Diaz A., Limon D.I., Mayoral M.A., Chanez-Cardenas M.E., Zenteno E., Montano L.F., Guevara J., Espinosa B. (2013). Amyloid-beta(25-35) induces a permanent phosphorylation of HSF-1, but a transitory and inflammation-independent overexpression of Hsp-70 in C6 astrocytoma cells. Neuropeptides.

[47] Choi J., Malakowsky C.A., Talent J.M., Conrad C.C., Carroll C.A., Weintraub S.T., Gracy R.W. (2003). Anti-apoptotic proteins are oxidized by Abeta25-35 in Alzheimer’s fibroblasts. Biochim. Biophys. Acta.

[48] Choi Y.J., Kim N.H., Lim M.S., Lee H.J., Kim S.S., Chun W. (2014). Geldanamycin attenuates 3nitropropionic acidinduced apoptosis and JNK activation through the expression of HSP 70 in striatal cells. Int. J. Mol. Med..

[49] Shabbir A., Bianchetti E., Cargonja R., Petrovic A., Mladinic M., Pilipovic K., Nistri A. (2015). Role of HSP70 in motoneuron survival after excitotoxic stress in a rat spinal cord injury model in vitro. Eur. J. Neurosci..

[50] Wiesneth S., Jurgenliemk G. (2017). Total phenolic and tannins determination: A modification of Ph. Eur. 2.8.14 for higher throughput. Pharmazie.

[51] Bieschke J., Russ J., Friedrich R.P., Ehrnhoefer D.E., Wobst H., Neugebauer K., Wanker E.E. (2010). EGCG remodels mature alpha-synuclein and amyloid-beta fibrils and reduces cellular toxicity. Proc. Natl. Acad. Sci. USA.

[52] Meng F., Abedini A., Plesner A., Verchere C.B., Raleigh D.P. (2010). The flavanol (-)-epigallocatechin 3-gallate inhibits amyloid formation by islet amyloid polypeptide, disaggregates amyloid fibrils, and protects cultured cells against IAPP-induced toxicity. Biochemistry.

[53] Porat Y., Abramowitz A., Gazit E. (2006). Inhibition of amyloid fibril formation by polyphenols: Structural similarity and aromatic interactions as a common inhibition mechanism. Chem. Biol. Drug Des..

[54] Jung H.A., Jung M.J., Kim J.Y., Chung H.Y., Choi J.S. (2003). Inhibitory activity of flavonoids from Prunus davidiana and other flavonoids on total ROS and hydroxyl radical generation. Arch. Pharmacal Res..

[55] Ruijters E.J., Weseler A.R., Kicken C., Haenen G.R., Bast A. (2013). The flavanol (-)-epicatechin and its metabolites protect against oxidative stress in primary endothelial cells via a direct antioxidant effect. Eur. J. Pharmacol..

[56] Shin H.A., Shin Y.S., Kang S.U., Kim J.H., Oh Y.T., Park K.H., Lee B.H., Kim C.H. (2014). Radioprotective effect of epicatechin in cultured human fibroblasts and zebrafish. J. Radiat. Res..

[57] Granado-Serrano A.B., Martin M.A., Bravo L., Goya L., Ramos S. (2010). Quercetin modulates NF-kappa B and AP-1/JNK pathways to induce cell death in human hepatoma cells. Nutr. Cancer.

[58] Kudin A.P., Malinska D., Kunz W.S. (2008). Sites of generation of reactive oxygen species in homogenates of brain tissue determined with the use of respiratory substrates and inhibitors. Biochim. Biophys. Acta.

[59] Huttemann M., Lee I., Malek M.H. (2012). (-)-Epicatechin maintains endurance training adaptation in mice after 14 days of detraining. FASEB J. Off. Publ. Fed. Am. Soc. Exp. Biol..

[60] Elbaz H.A., Lee I., Antwih D.A., Liu J., Huttemann M., Zielske S.P. (2014). Epicatechin stimulates mitochondrial activity and selectively sensitizes cancer cells to radiation. PLoS ONE.

[61] Jovaisaite V., Auwerx J. (2015). The mitochondrial unfolded protein response-synchronizing genomes. Curr. Opin. Cell Biol..

